# Characterization of a novel disease-associated mutation within *NPHS1* and its effects on nephrin phosphorylation and signaling

**DOI:** 10.1371/journal.pone.0203905

**Published:** 2018-09-13

**Authors:** C. James Cooper, Nikkita T. Dutta, Claire E. Martin, Tino D. Piscione, Paul S. Thorner, Nina Jones

**Affiliations:** 1 Department of Molecular and Cellular Biology, University of Guelph, Guelph, ON, Canada; 2 Division of Nephrology, The Hospital for Sick Children, Toronto, ON, Canada; 3 Department of Pathology and Laboratory Medicine, The Hospital for Sick Children and University of Toronto, Toronto, ON, Canada; University of Utah School of Medicine, UNITED STATES

## Abstract

Mutations in the transmembrane protein nephrin (encoded by *NPHS1*) underlie nearly half of all cases of congenital nephrotic syndrome (CNS), which is caused by aberrations in the blood filtering function of glomerular podocytes. Nephrin directly contributes to the structure of the filtration barrier, and it also serves as a signaling scaffold in podocytes, undergoing tyrosine phosphorylation on its cytoplasmic tail to recruit intracellular effector proteins. Nephrin phosphorylation is lost in several human and experimental models of glomerular disease, and genetic studies have confirmed its importance in maintenance of the filtration barrier. To date, however, the effect of CNS-associated *NPHS1* variants on nephrin phosphorylation remains to be determined, which hampers genotype-phenotype correlations. Here, we have characterized a novel nephrin sequence variant, A419T, which is expressed along with C623F in a patient presenting with CNS. Nephrin localization is altered in kidney biopsies, and we further demonstrate reduced surface expression and ER retention of A419T and C623F in cultured cells. Moreover, we show that both mutations impair nephrin tyrosine phosphorylation, and they exert dominant negative effects on wildtype nephrin signaling. Our findings thus reveal that missense mutations in the nephrin extracellular region can impact nephrin signaling, and they uncover a potential pathomechanism to explain the spectrum of clinical severity seen with mild *NPHS1* mutations.

## Introduction

Nephrotic syndrome is a kidney disorder with a wide range of etiologies and it is characterized by injury to the glomerular filtration barrier, resulting in leakage of essential blood proteins into the urine [1]. The glomerular filtration barrier is composed of an inner fenestrated endothelium, a glomerular basement membrane (GBM) and podocytes, which extend out over the surface of each capillary and form a meshwork of interdigitating actin-based foot processes [2]. Between adjacent foot processes, a sieve-like intercellular junction known as the slit diaphragm arises. Nephrin (encoded by *NPHS1*) is an essential structural component of the slit diaphragm, and mutations in this transmembrane protein lead to loss of foot process morphology (effacement) and proteinuria [3]. *NPHS1* was first identified in 1998 as the causative gene in congenital nephrotic syndrome (CNS) of the Finnish-type, which is inherited in an autosomal recessive manner [3]. Defined as the Fin^major^ mutation, this prototypic nonsense mutation in the nephrin ectodomain results in a complete loss of nephrin protein. Since this time, more than 250 different nonsense, frameshift, splice-site or missense mutations have been annotated for *NPHS1* in the Human Gene Mutation Database (http://www.hgmd.cf.ac.uk), and they are distributed across all exons [4]. Mutations have been classified as mild or severe, according to predicted effects on nephrin protein expression [5,6], and they variably impact the age of onset and disease progression [7]. Mutations in *NPHS1* account for ~40% of all cases of CNS [8]; therefore, understanding the functional consequences of each mutation has implications for genotype-phenotype correlations and clinical outlook.

In addition to the structural role of the nephrin ectodomain within the slit diaphragm, nephrin also functions as a signaling protein [9] undergoing tyrosine phosphorylation on its intracellular tail via the Src family kinase Fyn [10]. Anchored at the plasma membrane with podocin, phosphorylated nephrin promotes recruitment of key organizers of the podocyte actin cytoskeleton, such as Nck [11], as well as activation of transcription factors including AP-1 [12]. Tyrosine phosphorylation of nephrin is essential in mice for podocyte maintenance and restoration of injured foot processes [13] and this phosphorylation is reduced in human and experimental renal diseases [14,15]. To date, however, the tyrosine phosphorylation status of inherited nephrin variants and its impact on nephrin function is not well understood.

Here, we have characterized a novel nephrin sequence variant, A419T, which is expressed in a patient presenting with recurring nephrotic syndrome and in another family member with late onset glomerular disease. The patient is a compound heterozygote for the novel mutation and a previously characterized mutation, C623F, which is not properly expressed on the cell surface [5]. We demonstrate altered trafficking and tyrosine phosphorylation of A419T and C623F, and reveal dominant negative effects of both mutations on wildtype (WT) nephrin signaling. Our findings thus uncover a potential molecular mechanism by which mild mutations in nephrin can perturb filtration barrier integrity.

## Materials and methods

### Patients, molecular analysis and study approval

The proband underwent clinical assessment and renal biopsy for CNS, and three additional members of the family were ascertained. Blood was collected for DNA isolation from all participants after receiving informed written consent, in accordance with study approval from the Research Ethics Board at the Hospital for Sick Children. Genomic DNA was extracted using standard procedures, and subject to PCR amplification and direct sequencing of the entire *NPHS1* gene at the Genome Diagnostics Laboratory of the Hospital for Sick Children. Formalin-fixed paraffin embedded samples from biopsy material were obtained from the proband. *In silico* analysis of mutations was performed with PolyPhen-2 (http://genetics.bwh.harvard.edu/pph2/index.shtml) and SIFT (http://sift.bii.a-star.edu.sg) software.

### Plasmid*s*

Point mutations were introduced into Myc-tagged human nephrin cDNA in pcDNA3.1 [11] using site-directed mutagenesis with Pfx Platinum polymerase (Invitrogen) and confirmed by DNA sequencing. Plasmids encoding podocin, pGL3-AP-1 firefly luciferase and pRL-SV40-renilla luciferase were described previously [16]. Plasmids encoding myristoylated (plasma membrane targeted) GFP (pEGFP-N3 Myr-GFP) and pEYFP-ER were provided by Dr. George Harauz (University of Guelph).

### Antibodies

Primary antibodies used for these experiments were as follows: mouse anti-Myc (Cell Signalling, 9B11), mouse anti-phosphotyrosine 4G10 (#16–101; Upstate Biotechnology), rabbit anti-podocin (Sigma Aldrich, P0372) and mouse anti-β-actin (Sigma Aldrich, A1978). Rabbit anti-nephrin antibodies were provided by Dr. Tomoko Takano (McGill University) [10]. Phospho-specific anti-nephrin antibodies (Y1176/Y1193 and Y1217) were generated and validated previously [15]. Secondary antibodies (all Invitrogen) included horseradish peroxidase (HRP)-conjugated goat anti-rabbit (#A11008) and HRP-conjugated goat anti-mouse (#A11001), as well as goat anti-mouse Alexa Fluor-conjugated 594 (#A11005).

### Immunohistochemistry of human kidney tissue sections

Sections were cut at 4 microns from formalin-fixed paraffin embedded tissue blocks and processed in the Dako Omnis automated platform with the EnVision Flex System (Agilent Technologies, Santa Clara, CA). Antigen retrieval was performed at 97°C for 30 minutes in EnVision Flex Tris High pH buffer (Agilent). Nephrin antibody (Santa Cruz Biotechnology, #sc-376522, Santa Cruz, CA) was diluted 1:300 and applied for 30 minutes. Detection was for 30 minutes with the EnVision Flex HRP reagent (Agilent). Tissue sections were then counterstained with hematoxylin.

### Cell culture

Human embryonic kidney (HEK) 293T and COS-1 cell lines were obtained from the American Type Culture Collection (Manassas, VA, USA). Cells were cultured in Dulbecco’s Modified Eagle Medium (DMEM) (Sigma-Aldrich) supplemented with 10% Fetal Bovine Serum, 200 Units/mL penicillin and 200 μg/mL streptomycin (Invitrogen) and maintained at 37°C with 5% CO_2_. Transient transfection was performed using 1–5 μg of DNA with polyethyleneimine (PEI) for 24–48 hours.

### Cell lysis and Western blotting

Following transfection, cells were lysed in Phospholipase C (PLC)+ lysis buffer (50 mM Hepes [pH 7.5], 150 mM NaCl, 10% glycerol, 1% Triton X-100, 15 mM MgCl2, 1 mM EGTA, 10 mM NaPPi, and 100 mM NaF) supplemented with fresh protease and phosphatase inhibitors (1 mM PMSF, 1 mM sodium orthovanadate, 10 μg/ml aprotinin, and 10 μg/ml leupeptin) by vortexing and sonicating on ice. Proteins from cleared lysates were resolved via SDS-PAGE and transferred to PVDF membrane (Millipore). Membranes were blocked in TBST containing 5% nonfat milk powder or bovine serum albumin, and incubated overnight at 4°C with primary antibody. After washing, membranes were incubated with HRP-conjugated secondary antibody diluted at 1:10,000 in TBST for 1 hour at room temperature. Signals were detected using ECL Western blotting substrate (Pierce) or Luminata Crescendo (Millipore), and membranes were exposed to film (Pierce). Values used for densitometry were obtained using ImageLab v2.0 analysis software (BioRad).

### Cell surface biotinylation

Transfected cells were incubated with 5 ml of 1 mg/ml EZ-Link Biotin-X-NHS dissolved in borate buffer (10 mM boric acid, 150 mM NaCl [pH 8.0]) for 45 minutes at 4°C. Coupling was terminated by washing two times (2 minutes each) with 15 mM glycine in PBS at 4°C. After a final wash in PBS, cells were lysed in PLC+ buffer, and sonicated for 10 seconds. Lysates were then subjected to streptavidin agarose precipitation overnight at 4°C or resuspended in 2xSDS loading buffer to act as the input loading control. After overnight precipitation, beads were washed three times successively in PLC+, and spun at 3000 × *g* for 1 minute between washes before finally being resuspended and boiled in 50 μl of 2xSDS loading buffer. Surface nephrin in each sample was determined by dividing the density of band observed in immunoblotting of the precipitated sample by the density of band observed in the input sample. The ratio of this value in test samples to the value obtained in control samples was calculated and represents the amount of surface nephrin in indicated test groups relative to control.

### Fluorescence immunostaining

Cells were seeded on coverslips in 6-well plates at 100,000 cells per well, and transfected with 2 μg of Myc-tagged nephrin plasmids along with 1μg of pEGFP-N3 Myr-GFP or pEYFP-ER. 24–48 hours post-transfection, cells were fixed in 4% paraformaldehyde (PFA) for 10–15 minutes, followed by permeabilization with 0.2% Triton X-100 in PBS for 10 minutes. Slides were blocked in 10% normal goat serum in PBS for 1 hour then incubated at room temperature for 1 hour in 1:200 dilution of primary antibody in blocking solution. Following washing in PBS, secondary antibody was added at a 1:400 dilution in blocking solution for 30 minutes. After a final series of washes, slides were mounted in ProLong Gold AntiFade reagent containing DAPI (Invitrogen). Slides were imaged using a Leica DM6000 confocal laser scanning microscope coupled to a Leica TCS SP5 platform (University of Guelph Molecular and Cellular Imaging Facility). For 3D digital reconstruction, image sets of 0.3 micron optical serial sections were acquired and analyzed using ImageJ software (National Institutes of Health).

### Dual luciferase reporter assay

HEK293T cells were transiently transfected with 1 μg AP1 firefly luciferase, 80 ng renilla luciferase and additional plasmids as indicated, to a total DNA amount of 4 μg per 35-mm dish. Cells were collected on ice in 1 ml PBS, centrifuged at 4,000 rpm at 4°C for 2 minutes, lysed in 300 μl of 1× passive lysis buffer (Promega), and spun again at 13,000 rpm at 4°C for 1 minute to remove insoluble material. Luciferase activity was measured using Dual-Luciferase Reporter Assay System (Promega) on a POLARstar Omega microplate reader (BMG Labtech) and normalized to renilla activity to correct for transfection efficiency. Protein expression was confirmed by immunoblot.

### Statistical analysis

Data are reported as arithmetic means ± SEM. Data analysis was performed using Prism (version 5, GraphPad, Carlsbad, USA). Statistical significance was determined with the Kruskal-Wallis test with selected Dunn’s multiple comparisons *post-hoc*. For all analyses, a *P*-value < 0.05 was considered statistically significant.

## Results

### Identification of a novel *NPHS1* transition mutation in congenital nephrotic syndrome

**Fig 1:**
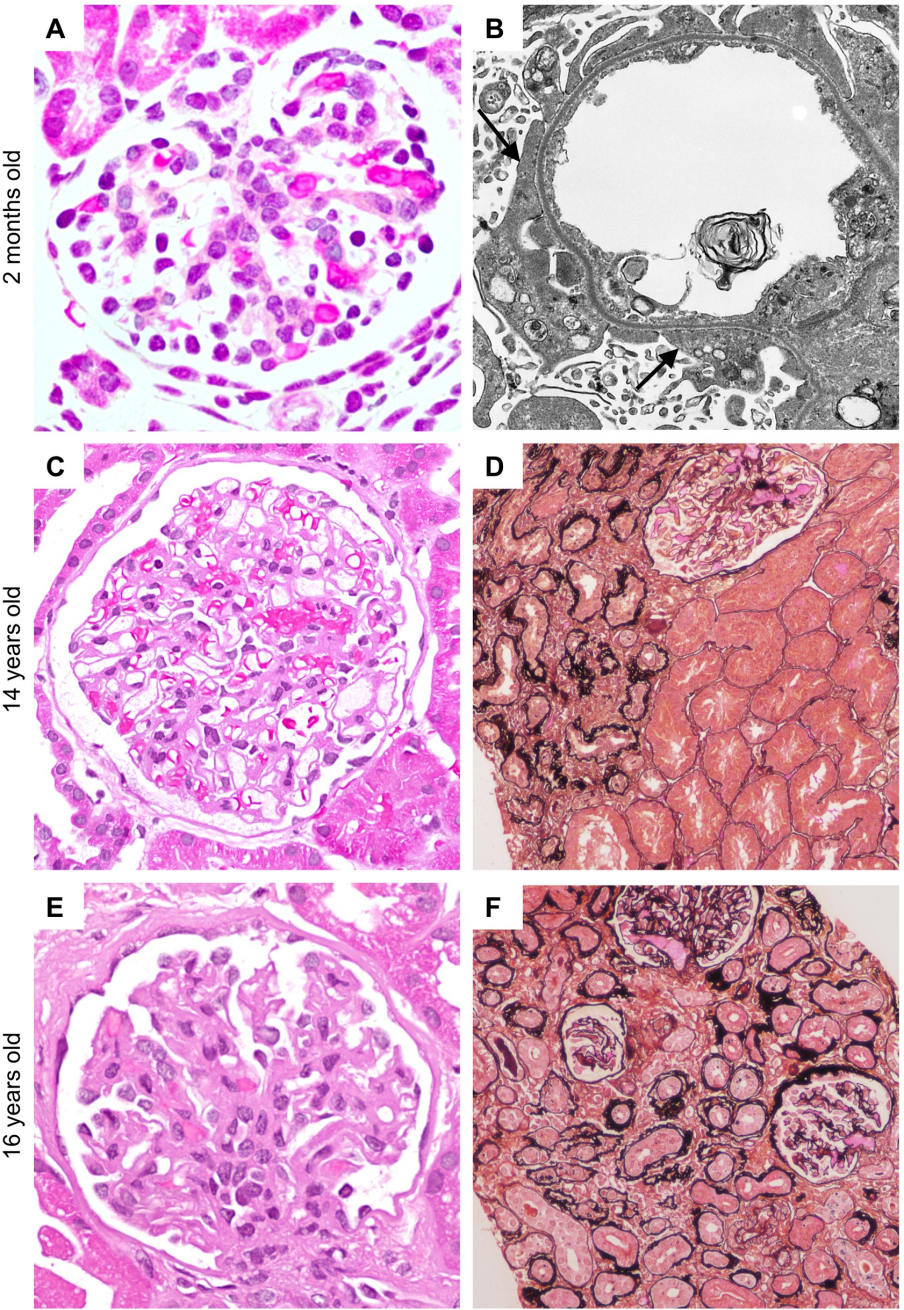
Microscopic appearance of the kidney in the proband. At 5 weeks of age, the glomeruli appeared normal by light microscopy (A) but by electron microscopy, there is widespread effacement of podocyte foot processes (arrows) (B). By 14 years of age, glomeruli still appear within normal limits (C) but there is focal tubular atrophy and interstitial fibrosis (upper left region) (D). By 16 years of age, glomeruli are becoming more consolidated with reduced capillary loops (E) and there is moderate tubular atrophy and interstitial fibrosis (F). (A: hematoxylin phloxine saffron stain, original magnification x600; B: original magnification x2500; C and E: hematoxylin and eosin stain, original magnification x400; D and F: periodic acid ammoniacal silver stain, original magnification x200).

**Fig 2:**
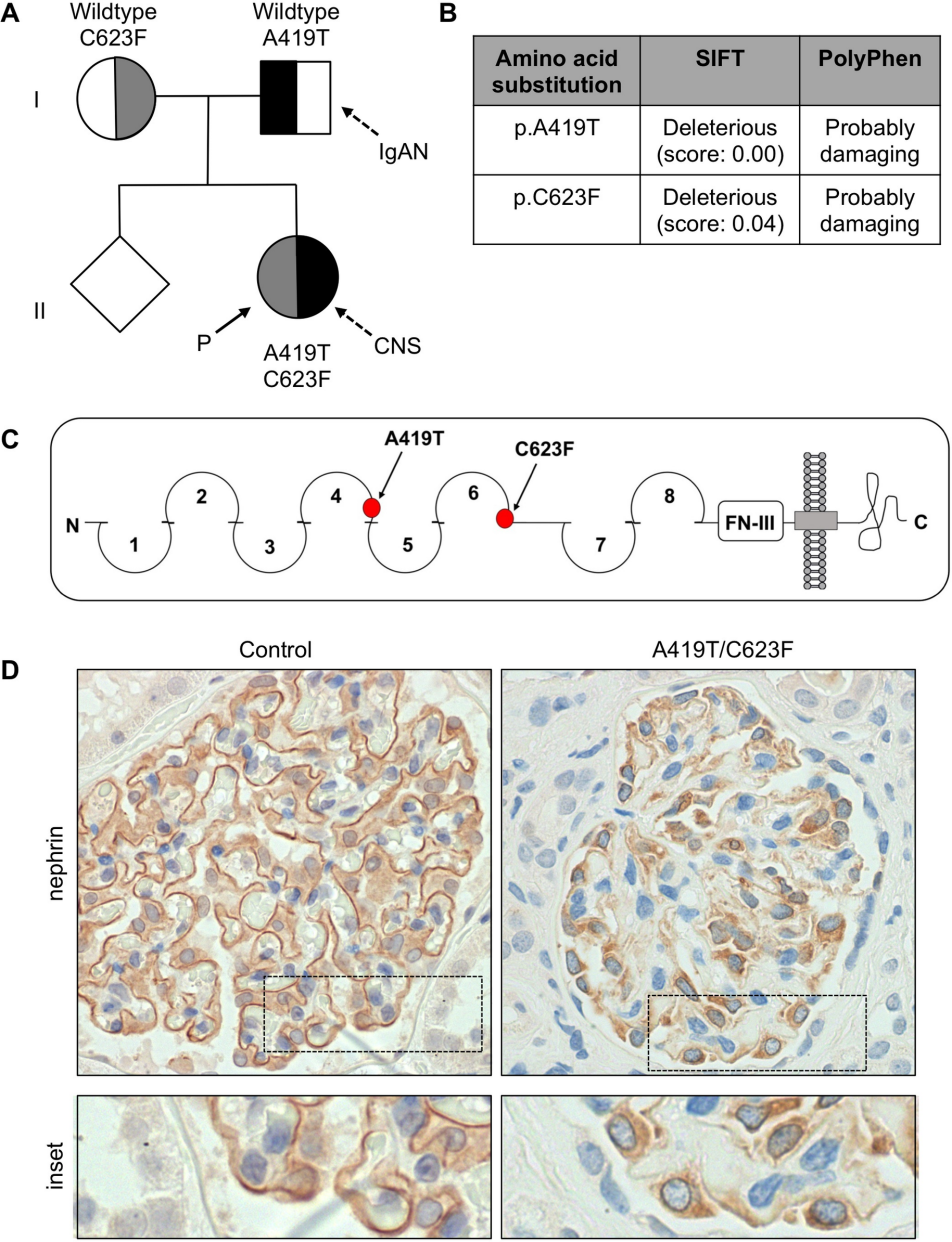
Family pedigree, mutation analysis and nephrin immunostaining. (A) Pedigree depicting the proband (P, solid arrow) as a compound heterozygote for inherited A419T and C623F mutations in *NPHS1*. The parents are obligate carriers of each mutation, and the sibling is mutation negative. (B) Functional consequences of A419T and C623F were assessed using protein structure prediction algorithms SIFT (Sorting Intolerant From Tolerant) and PolyPhen (Polymorphism Phenotyping). (C) Schematic representation of nephrin protein and location of mutations under investigation. (D) Immunoperoxidase staining for nephrin in renal biopsies taken from normal control 16-year-old, and the proband at 16 years of age. Compared to the normal control, the proband’s biopsy shows incomplete staining of nephrin along the GBM, with positive staining for nephrin within the podocyte cell bodies. D: original magnification x600. CNS, congenital nephrotic syndrome; IgAN, IgA nephropathy.

The proband presented with CNS at 2 months of age, with recurrent episodes at age 14 and age 16 following renal transplantation. Biopsies taken at all 3 ages show progressive glomerular changes (Fig 1A, 1C and 1E) and tubular atrophy (Fig 1D and 1F), with widespread podocyte foot process effacement at first presentation (Fig 1B). Mutation analysis was performed using PCR and direct sequencing of *NPHS1*. Biallelic missense mutations were identified within exon 10 (c.1255G>A, p.A419T) and exon 14 (c.1868G>T, p.C623F), and were individually detected within the proband’s mother and father, but not in a sibling (Fig 2A). IgA nephropathy (IgAN) was later revealed in the father. Protein function prediction algorithms identified both A419T and C623F as deleterious and probably damaging (Fig 2B). C623F likely disrupts formation of the disulfide bond forming the sixth Ig-like domain (Fig 2C) and has previously been shown to impact nephrin surface expression [5]. Similarly, the A419T mutation lies adjacent to the cysteine-based disulfide bond formed by C417 within the fourth Ig-like domain, suggesting that it may also disrupt protein folding and nephrin trafficking (Fig 2C). To examine nephrin distribution, anti-nephrin antibody staining was performed on renal biopsies obtained from the proband and an age-matched normal control, as well as from a patient harbouring a nephrin-null mutation to verify antibody specificity (S1 Fig). Compared to the normal control, incomplete staining of nephrin was observed along the GBM, with aggregation of nephrin within the podocyte cell bodies (Fig 2D). As the phenotype of A419T has not yet been characterized, we pursued its effects on nephrin signaling alongside C623F.

### Generation and surface expression of nephrin A419T missense mutation

**Fig 3:**
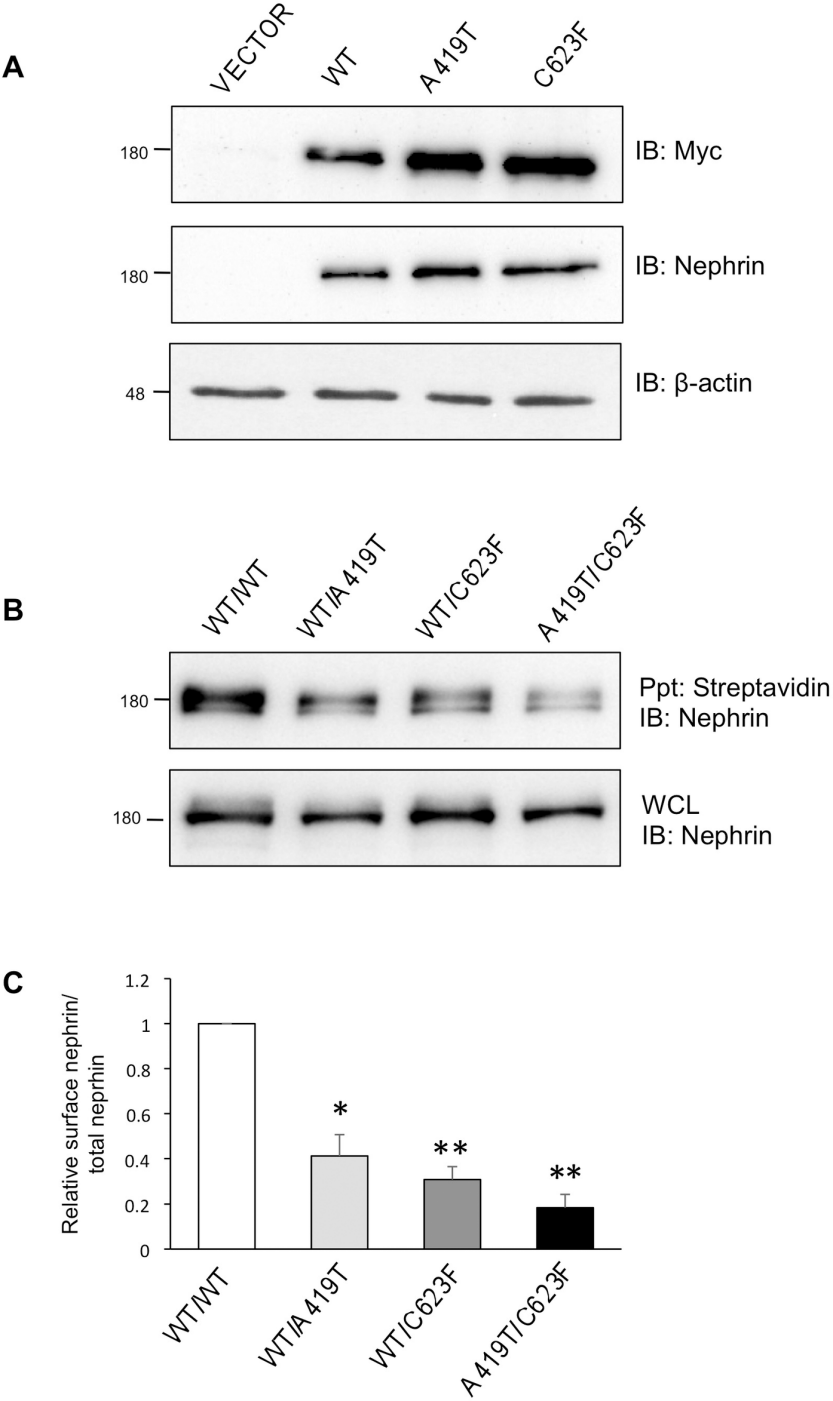
Decreased surface expression of missense mutations. (A) HEK293T cells were transfected with Myc-tagged nephrin variants or vector alone, and lysates were immunoblotted (IB) with anti-Myc and anti-nephrin to show comparable expression of A419T and C623F to wildtype (WT), or β-actin as loading control. (B) HEK293T cells with compound expression of nephrin variants (as indicated) were subject to surface biotinylation, followed by lysis, streptavidin precipitation (Ppt) and nephrin immunoblotting (IB). A portion of the initial whole cell lysate (WCL) was saved to indicate equivalent input. (C) Densitometric quantitation (n = 4) of streptavidin-precipitated biotinylated nephrin (surface) compared with total nephrin (input) from (B), showing a reduction in surface nephrin levels with A419T and/or C623F mutations. All values were made relative to surface nephrin levels in WT/WT samples. *P < 0.05; **P < 0.01; by Kruskal-Wallis test with *post-hoc* Dunn’s multiple comparison tests as compared to WT/WT.

To investigate whether this novel missense mutation might affect nephrin function, A419T was introduced into Myc-tagged full-length human wildtype (WT) nephrin. We also engineered C623F, to study the effects of the biallelic mutations present within the index patient. Both nephrin mutants were expressed in HEK293T cells at levels similar to those of the WT protein (Fig 3A). To next examine whether A419T could impact nephrin expression on the cell surface, biotinylation assays were performed in HEK293T cells. Cells co-expressing either A419T or C623F with WT nephrin show a significant reduction in surface nephrin expression compared to cells expressing WT alone, while cells co-expressing A419T with C623F show an even further decrease (Fig 3B). Densitometric quantitation indicates that less than half of the amount of nephrin is present on the surface with the A419T or C623F variants, and this drops to less than 20% of WT levels with A419T and C623F (Fig 3C).

### Impaired localization of nephrin A419T at the plasma membrane

**Fig 4:**
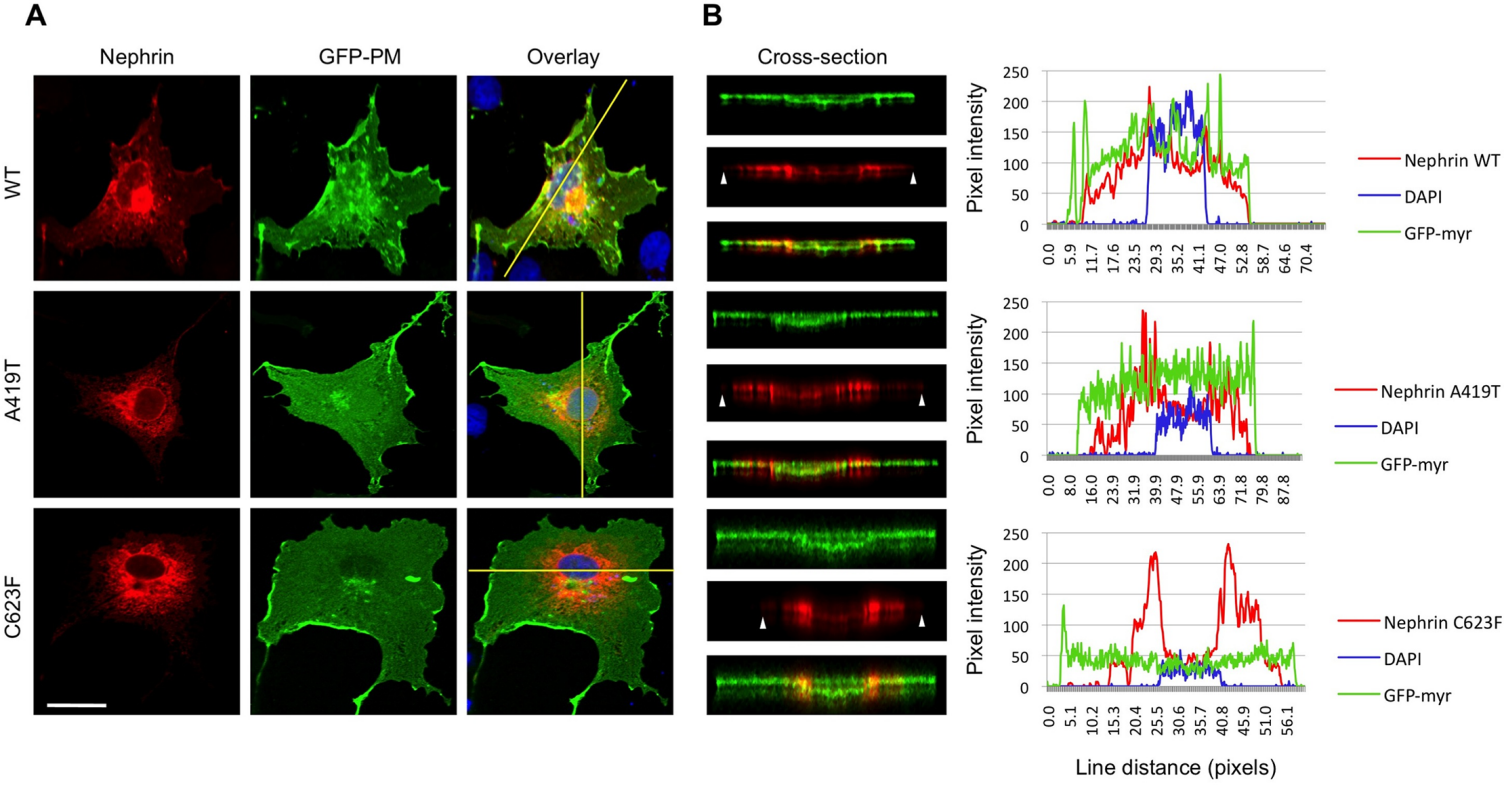
Altered cellular trafficking of nephrin mutants A419T and C623F to the plasma membrane. (A) Fluorescent confocal micrographs of COS-1 cells co-expressing Myc-tagged nephrin variants (red) and myristoylated-GFP as plasma membrane (PM) marker (green), counterstained with DAPI (blue) to highlight nuclei. (B) 3D digital reconstruction cross-sections and line scan analyses (regions of interest indicated with yellow lines in overlays in A) were used to score intracellular distribution of nephrin relative to the GFP-PM marker. Intensity plots show wildtype (WT) nephrin is well distributed across all regions of the cytosol, while A419T and C623F mutants appear to aggregate in the perinuclear region, with reduced (A419T) or near absent (C623F) expression at the PM (white arrows).

**Fig 5:**
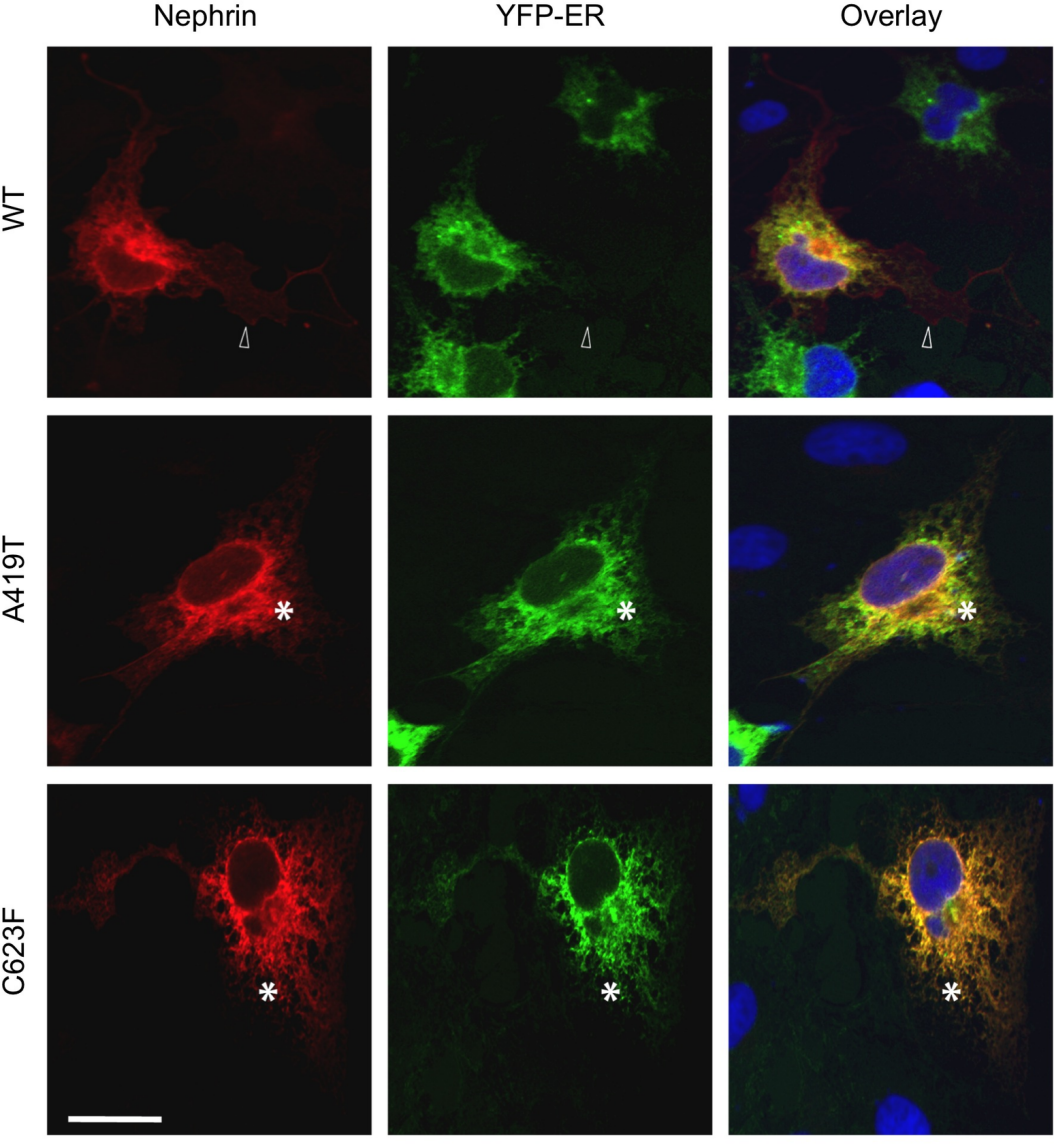
Retention of nephrin mutants A419T and C623F within the endoplasmic reticulum. Fluorescent confocal micrographs of COS-1 cells co-expressing Myc-tagged nephrin variants (red) and EYFP-tagged endoplasmic reticulum (ER) marker (green), counterstained with DAPI (blue) to highlight nuclei. Merged images show substantial co-localization of the ER marker with nephrin mutants A419T and C623F, as indicated by asterisks. Wildtype (WT) nephrin shows a more dispersed pattern into the cell periphery, as indicated by hollow arrow in upper panel.

Previous studies have demonstrated that C623F preferentially accumulates in the endoplasmic reticulum (ER) [5], thus we next assessed the cellular localization of A419T via dual immunofluorescence confocal microscopy. Myc-tagged WT, A419T and C623F nephrin variants (red) were expressed in COS-1 cells (to facilitate imaging), in conjunction with plasma membrane-targeted GFP (green). WT nephrin consistently distributed to the plasma membrane, but similar to C623F, A419T appeared to be enriched in a perinuclear compartment consistent with the ER (Fig 4A). To examine this in further detail, we obtained cross-sectional micrographs of 3D digital reconstructions from confocal z-axis slices and performed line scan analysis. WT nephrin is present across all regions of the cell, and line graphs indicate robust colocalization of WT nephrin with the plasma membrane marker (Fig 4B, top panels). By contrast, C623F is restricted to the region surrounding the nucleus, and line graphs highlight the near absence of nephrin signal at the cell periphery (Fig 4B, bottom panels). A419T appears to show an intermediate phenotype, with enhanced perinuclear accumulation and reduced diffusion of nephrin to the outer region of the cell (Fig 4B, middle panels). Using a fluorescent ER marker, we confirmed that the 2 mutants colocalize with the ER, whereas the WT is distributed broadly throughout the cell (Fig 5).

### Impact of A419T on nephrin phosphorylation and downstream signalling

**Fig 6:**
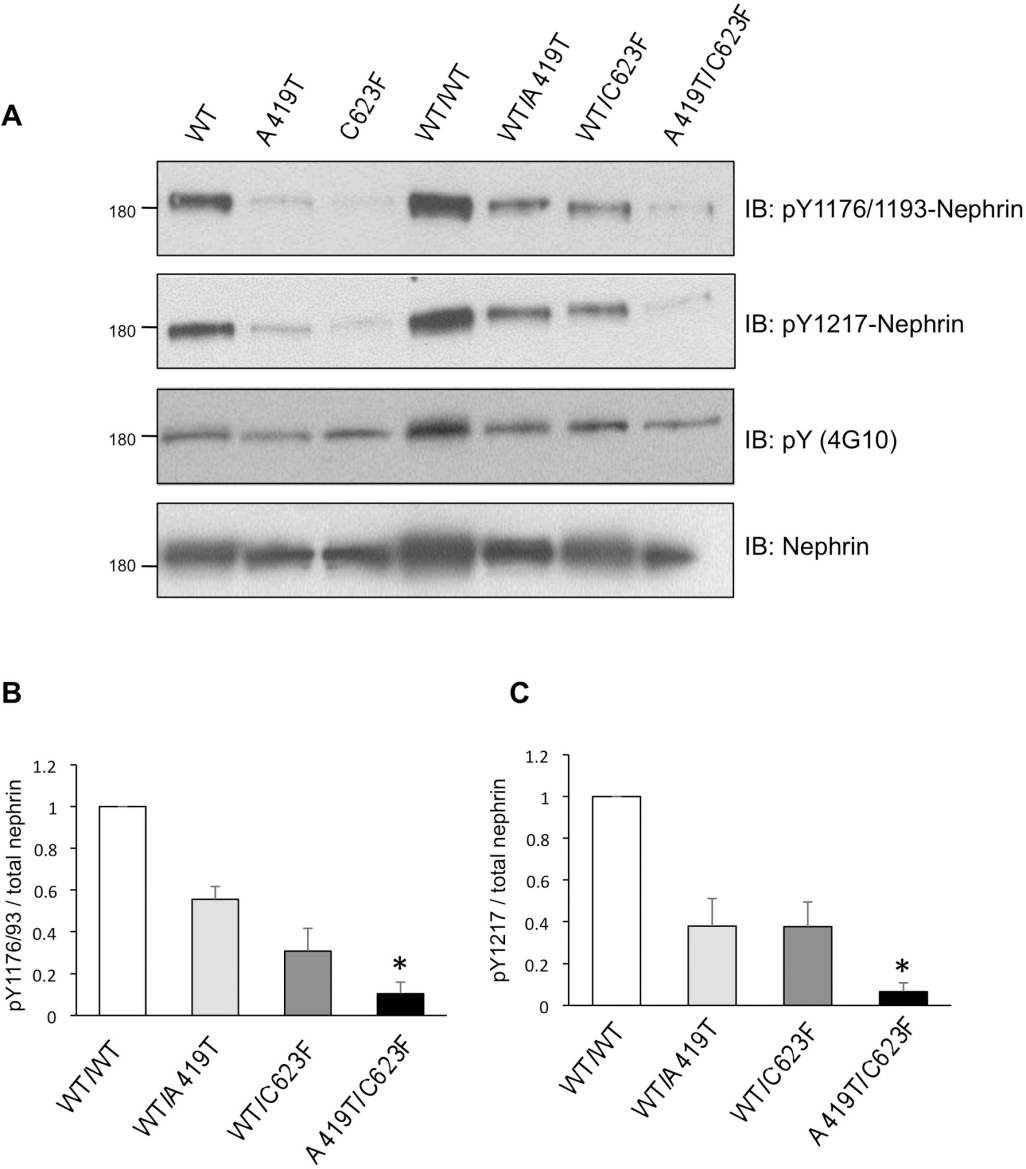
Reduced nephrin tyrosine phosphorylation with A419T and C623F mutations. (A) HEK293T cells were transfected with Myc-tagged nephrin variants, alone or in combination, and lysates were immunoblotted (IB) as indicated with antibodies to nephrin, total phosphotyrosine (pY) or site-specific phosphorylated nephrin (pY1176 or pY1217). A419T and C623F show reduced nephrin tyrosine phosphorylation compared to wildtype (WT). Coexpression of either mutant with WT reduces overall nephrin phosphorylation, and this phosphorylation is barely detected upon coexpression of both mutants. (B,C) Densitometric analyses of immunoblot intensities in lanes 4–7 are expressed as a ratio of phospho-nephrin/total nephrin relative to WT/WT. *P < 0.05; by Kruskal-Wallis test with *post-hoc* Dunn’s multiple comparison tests as compared to WT/WT.

Localization of nephrin at the cell periphery is required for its tyrosine phosphorylation and downstream signaling, thus we next investigated the functional consequences of reduced surface expression of the nephrin mutants. A419T and C623F were expressed in HEK293T cells, and overall tyrosine phosphorylation, as well as site-specific phosphorylation at tyrosine residues 1176/1193 and 1217, was compared to WT. Despite equal expression of total nephrin, both mutants displayed a substantial decrease in nephrin phosphorylation which corresponded to the 3 key signaling residues (Fig 6A, lanes 1–3). Next, we assessed the effects of biallelic expression of the mutants together (as in the proband) or with WT nephrin (as might be presumed in the parent carriers). Co-expression of either mutant with WT nephrin significantly reduced overall and site-specific nephrin tyrosine phosphorylation (Fig 6A, lanes 4–6), and densitometry revealed this to be less than 50% of that observed with WT/WT (Fig 6B and 6C), suggesting a dominant negative effect of these mutations on the WT protein. Nephrin tyrosine phosphorylation on Y1176/Y1193 and Y1217 was almost completely lost with compound expression of A419T and C623F (Fig 6A, lane 7), with less than 10% of site-specific phosphorylation remaining (Fig 6B and 6C).

**Fig 7:**
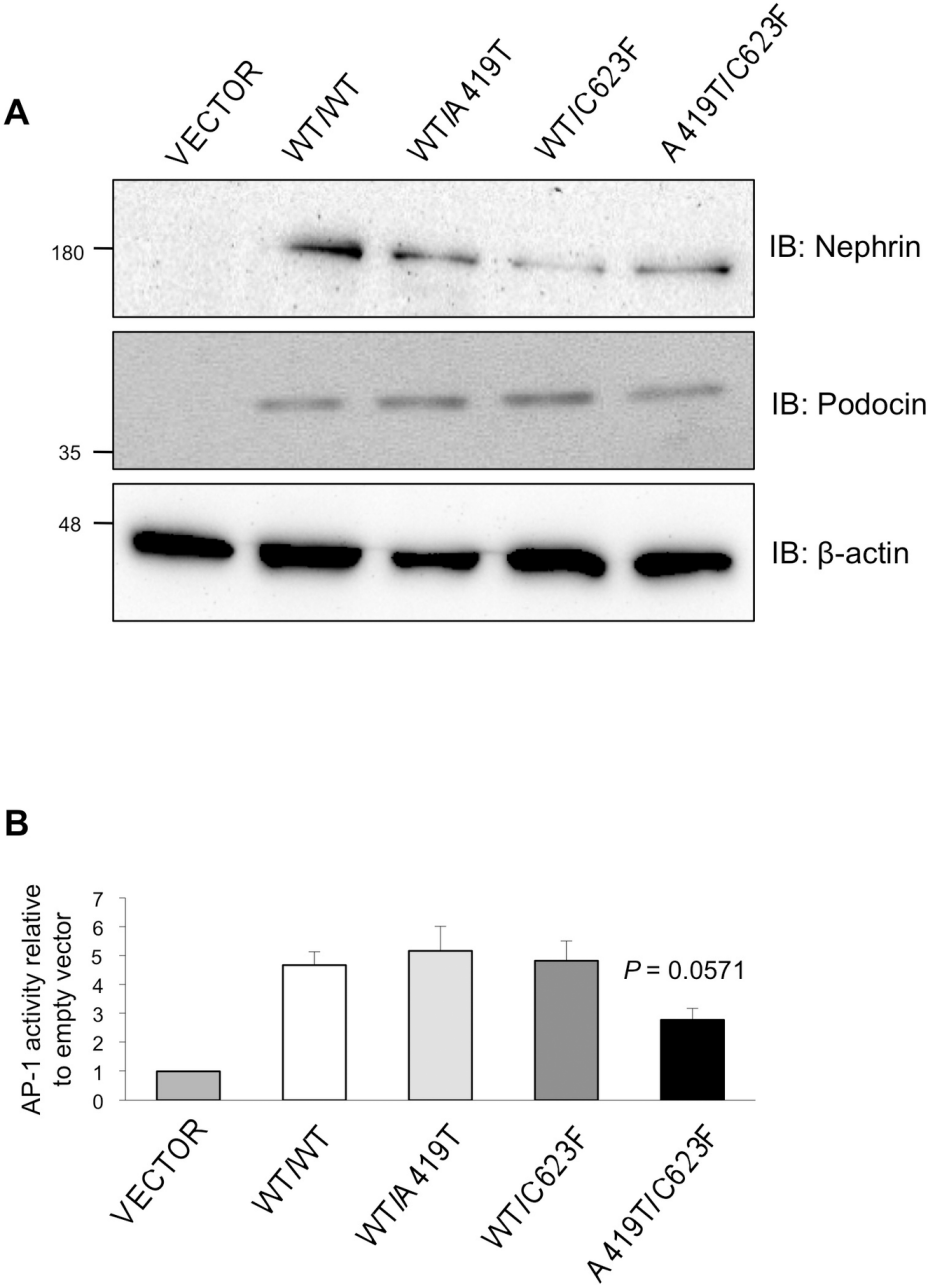
Compound expression of A419T and C623F compromises nephrin downstream signaling. (A) HEK293T cells were transfected with Myc-tagged nephrin variants and podocin, in addition to AP-1 firefly and renilla luciferase reporters. Lysates were immunoblotted (IB) to confirm equal protein expression with β-actin levels serving as loading control. (B) Lysates from (A) were further subjected to luciferase assay, and total activity of renilla was used to normalize expression. A419T and C623F induce AP-1 activation with WT nephrin at a level comparable to WT alone, but this effect is reduced (*P* = 0.0571) upon coexpression of both mutants (as assessed by Kruskal-Wallis test with *post-hoc* Dunn’s multiple comparison tests compared to WT/WT).

Lastly, we monitored activation of the AP-1 transcription factor by the nephrin mutants, as this is a well-established downstream target of nephrin signaling [12,16]. HEK293T cells were transfected with WT, A419T and/or C623F or vector alone in addition to plasmids encoding podocin, AP-1 luciferase and renilla luciferase (as a transfection control). Lysates were then subject to immunoblot assay to confirm equal protein expression (Fig 7A) or luminescence detection. Expression of WT nephrin increased AP-1 promoter activity 5-fold over baseline (Fig 7B). Co-expression of A419T or C623F with WT nephrin activated the AP-1 promoter to the same degree as WT nephrin alone; however, their compound expression impaired AP-1 activation (Fig 7B). Altogether, these results suggest that the A419T and C623F variants function as mild yet deleterious mutations.

## Discussion

In this study, we have characterized the functional properties of A419T and C623F nephrin mutations. Both variants can be expressed as full-length proteins, though they demonstrate reduced membrane targeting and ER retention, consistent with the majority of nephrin missense mutations analyzed to date [5]. Moreover, both mutations reduce nephrin tyrosine phosphorylation and downstream signaling, and they display a dominant negative effect on phosphorylation of the WT protein, revealing an important mechanism for their pathogenicity.

Nephrin is the core protein present within the slit diaphragm, thus reductions in its surface expression compromise the structural integrity of the filtration barrier. Indeed, mutations in *NPHS1* occur in nearly half of all infants with CNS [17], and they display varying degrees of ER retention. Most mild mutations present as compound heterozygosity, as described herein, where an individual is heterozygous for 2 *NPHS1* mutations, or through digenic inheritance, where dual mutations in *NPHS1* and *NPHS2* may arise [6]. Of note, *NPHS1* mutations have been identified that appear to cause disease in later adolescence [7] through to early adulthood [18]. Downregulation of nephrin has similarly been documented in many forms of acquired glomerular diseases, such as diabetic nephropathy [19–23], minimal change disease (MCD) [24,25], focal segmental glomerulosclerosis (FSGS) [26], membranous nephropathy [26,27], and others including IgAN, where reductions in *NPHS1* expression correlate with increased proteinuria [28]. Together these studies lead to the suggestion that, beyond the perinatal window, declining nephrin levels can likewise promote glomerular damage. In support of this, a recent study in mice demonstrated that prolonged knockdown of nephrin expression can induce mild disease in adult animals, and disease could be exacerbated by nephrin suppression in experimental injury models, even after only short-term disruption of nephrin expression [29]. Our findings extend this insight to demonstrate that mild nephrin mutations can also compromise functions of the normal nephrin protein. Moreover, it is tempting to speculate that carriers of single *NPHS1* mutations, as in the case of the father herein with adult-onset IgAN, may be predisposed to kidney diseases that manifest later in life, though we cannot rule out the possibility of undetected mutations in other key podocyte genes.

Nephrin not only serves as the physical barrier of the slit diaphragm, but also as a signaling platform due to its extensive network of cytoplasmic binding partners [30]. These include the Nck family of actin adaptors, which associate with tyrosine residues 1176, 1193 and 1217 on the cytoplasmic tail of nephrin [11,31,32] and further promote nephrin tyrosine phosphorylation [33]. Several endocytic adaptor proteins are also recruited to these tyrosines, and they differentially modulate nephrin turnover on the cell surface [16,34,35]. Mutations in the cytoplasmic region of nephrin have been reported with varying clinical severity. The Fin^minor^ R1109X mutation, in which the majority of the tail is not expressed, results in CNS comparable to the Fin^major^ mutation where the entire nephrin protein is lost [3,36]. By contrast, the *NPHS1* mutation R1160X, which creates a smaller truncation yet similarly lacks Y1176, Y1193 and Y1217, causes childhood disease that is delayed and less severe compared to R1109X [6]. These phenotypes are paralleled in mouse studies where total nephrin knockout results in neonatal lethality with massive proteinuria [37], while site-specific disruption of nephrin Y1176, Y1193 and Y1217 (nephrin-Y3F mice) induces juvenile onset proteinuria with foot process effacement [13]. Nephrin-Y3F mice further display a reduced capacity to repair injured podocytes [13], and changes in phosphorylation of these tyrosines are associated with mislocalization of nephrin and proteinuria [16]. In our study, both A419T and C623F are not properly trafficked to the cell surface, which likely explains their reduced phosphorylation, though both of these variants induce a dominant negative effect on WT nephrin. Paradoxically however, this residual phosphorylation appears to be at a threshold sufficient to activate normal levels of AP-1 transcription, though the effect on other signaling outputs remains to be determined. We propose that mild mutations in *NPHS1* impacting nephrin localization and signaling could lead to progressive decline in the integrity of the glomerular filtration barrier.

As *NPHS1* mutations in Y1176, Y1193 or Y1217 have not been directly implicated in instances of human disease, a significant role for nephrin tyrosine phosphorylation in the pathogenesis of CNS has been largely overlooked. Hundreds of *NPHS1* mutations have now been annotated in CNS patients [4], particularly within hot-spots in the extracellular Ig-like motifs or their linker regions. However, not all mutations cause retention of nephrin in the ER of cultured cells, despite their classification as pathogenic using protein folding prediction algorithms, and their impact on nephrin tyrosine phosphorylation remains to be determined [38]. In this regard, a sole report characterizing the *NPHS1* V822M variant associated with relapsing MCD showed that this mutant could properly express on the cell surface, but it was defective in trafficking from the membrane and in its tyrosine phosphorylation kinetics [39]. Our findings support the notion that defective nephrin signaling may weaken the filtration barrier, and contribute to podocyte injury. With the availability of phospho-specific nephrin antibodies [15,32], it would be prudent to re-evaluate deleterious mutations that have been classified as mild, to assess their tyrosine phosphorylation status. Such genotype-phenotype correlations are necessary to improve clinical predictions. Furthermore, previous attempts have been made to restore expression of mutant nephrin proteins on the surface using a chemical chaperone approach [38], and our studies suggest that preserving nephrin phosphorylation may represent a novel therapeutic strategy for glomerular disease.

## Supporting information

S1 Fig Verification of nephrin antibody specificity in kidney sections. Immunoperoxidase staining for nephrin in renal biopsies obtained from a CNS case with a nephrin-null mutation (B) and an age-matched positive control (A), along with the proband at 16 years of age (C). No staining is observed with the nephrin-null mutation, while the normal control shows positive nephrin staining along the basement membrane. In the proband, nephrin staining along the basement membrane is weak and interrupted. The arrows mark podocytes with negative (A) or positive (C) peri-nuclear staining.
(TIF)
