# Supplementary figures and images for: Characterization of a novel disease-associated mutation within NPHS1 and its effects on nephrin phosphorylation and signaling
Source: PLoS One. 2018 Sep 13;13(9):e0203905. doi: 10.1371/journal.pone.0203905 (PMC6136785; doi:10.1371/journal.pone.0203905)

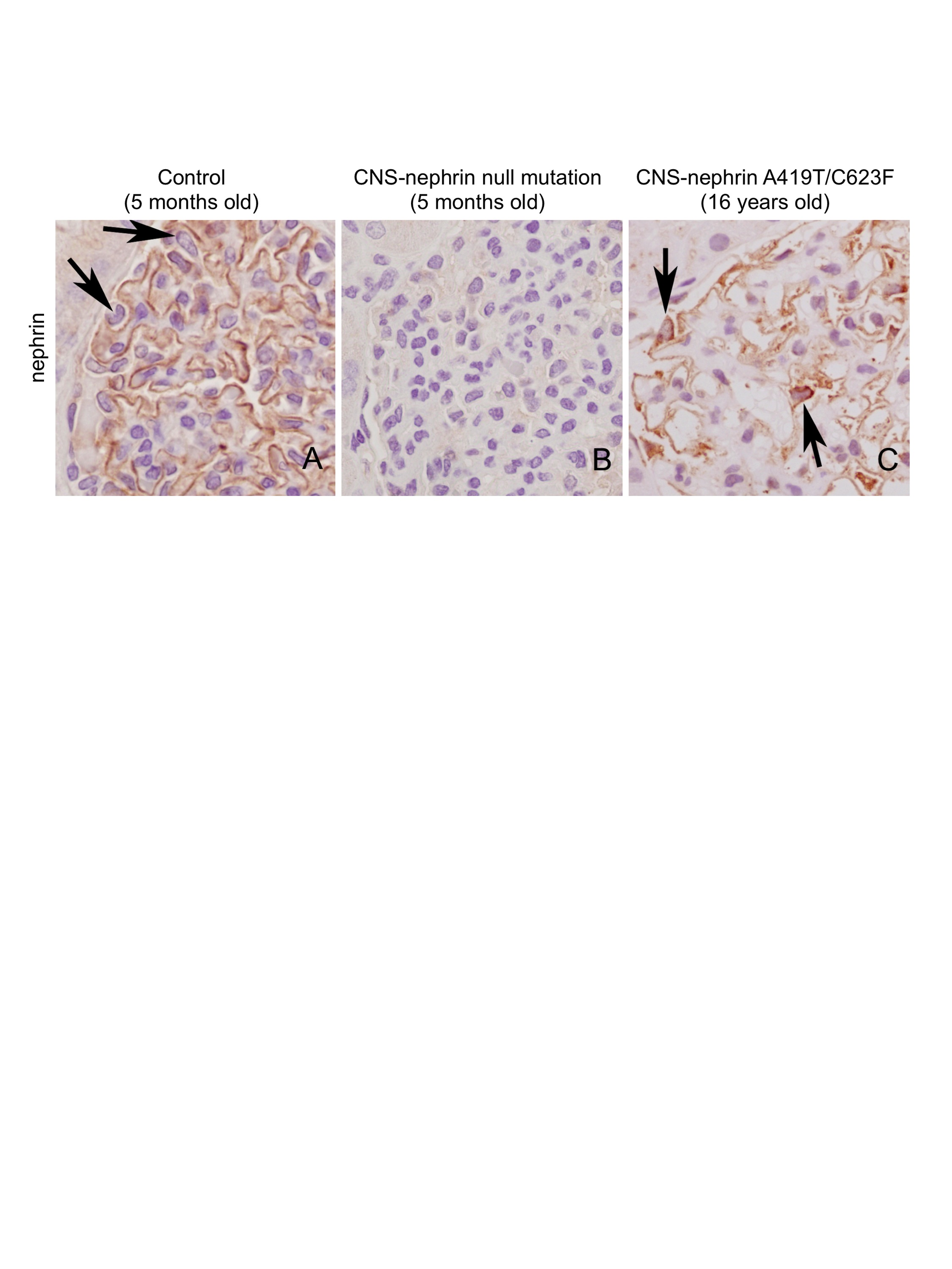

Supplement: S1 Fig — Immunoperoxidase staining for nephrin in renal biopsies obtained from a CNS case with a nephrin-null mutation (B) and an age-matched positive control (A), along with the proband at 16 years of age (C). No staining is observed with the nephrin-null mutation, while the normal control shows positive nephrin staining along the basement membrane. In the proband, nephrin staining along the basement membrane is weak and interrupted. The arrows mark podocytes with negative (A) or positive (C) peri-nuclear staining. (TIF) [file pone.0203905.s001.tif]
